# Case Report: Concomitant Diagnosis of Plasma Cell Leukemia in Patient With JAK2 Positive Myeloproliferative Neoplasm

**DOI:** 10.3389/fonc.2020.01497

**Published:** 2020-08-27

**Authors:** Christine J. Kurian, Colin Thomas, Sarah Houtmann, Thomas Klumpp, Adam Finn Binder

**Affiliations:** ^1^Department of Internal Medicine, Thomas Jefferson University Hospital, Philadelphia, PA, United States; ^2^Department of Medical Oncology, Thomas Jefferson University Hospital, Philadelphia, PA, United States

**Keywords:** case report, plasma cell leukemia, myeloproliferative neoplasm, JAK2 mutation, essential thrombocytosis

## Abstract

Plasma cell dyscrasias and myeloproliferative neoplasms (MPN) are hematologic malignancies arising from two distinct hematopoietic cell lineages. They rarely occur concomitantly. Here, we report a case of a patient with a recent diagnosis of a JAK2 V617F positive MPN who presented with a new diagnosis of plasma cell leukemia. The patient had presented to the hospital with a leukocytosis predominantly comprised of plasma cells, followed by work-up involving peripheral blood flow cytometry, FISH analysis, and bone-marrow biopsy. FISH analysis was suggestive of a common progenitor cell for these distinct hematologic malignancies. To our knowledge, this case represents the second reported instance of a concomitant JAK2 positive MPN with primary plasma cell leukemia.

## Introduction

There are several published reports of patients with concomitant myeloproliferative neoplasms (MPN) and plasma cell dyscrasias (PCD), the peculiarity of which being that these two malignancies arise from separate lineages in the hematopoietic ancestral tree (1, 2). Apart from case reports of patients with MPNs and PCDs, one prospective study found the coexistence of monoclonal gammopathy of undetermined significance (MGUS) in patients with a MPN to be 8.2%, which is higher than the average prevalence of MGUS in the general population (3%) (3, 4).

Other studies, however, have not found a significantly higher proportion of MGUS in patients with MPN—overall clouding the picture of this association (4). When patients are diagnosed with plasma cell dyscrasia and an MPN, based on one case series, it seems the MPN either precedes or is diagnosed concurrently with the plasma cell dyscrasia (5). Although bone marrow fibrosis is common on diagnosis of MM, ~38% of patients, it is likely a reactive process (5). There have been a few reports of myelofibrosis in plasma cell leukemia; however, these have been presumed to be reactive with negative JAK2 mutations (5, 6).

Unlike MGUS, smoldering myeloma, or multiple myeloma, primary plasma cell leukemia (pPCL) has a distinct pathogenesis (7). pPCL is defined as de novo leukemia having >2 × 10^9^/L plasma cells in the peripheral blood or plasma cells making up >20% of peripheral blood leukocytes. It is considered the most aggressive of all plasma cell neoplasms with poor overall survival (8).

Candoni et al. described a similar case in 2004 with a case of plasma cell leukemia occurring in a patient with thrombocythemia. At the time of his ET diagnosis, he was treated with aspirin, hydroxyurea, and busulphan. In this patient's PCL, there was a high expression of P-glycoprotein and notably his karyotype showed a deletion of chromosome 7 typically associated with secondary leukemias. It was thought that his previous cytoreductive therapy exposure with hydroxyurea and busulphan may have contributed to the development of PCL (9).

Here, we report a another case of a patient with JAK2 V617F myeloproliferative syndrome, most likely essential thrombocytosis (ET), and new diagnosis of plasma cell leukemia (PCL): this is the second known reported case in the literature.

## Case Description

A 76-year-old Caucasian woman with a history of paroxysmal atrial fibrillation, hypertension, chronic sciatica requiring assistance with a walker, gastroesophageal reflux disease, and a presumed diagnosis of JAK2 V617F positive ET initially presented to an outside hospital with a left clavicular fracture following a mechanical fall. The patient was incidentally found to have a leukocytosis of 71,000 cells/mcL (66% lymphocytic). She was transferred to Thomas Jefferson University Hospital (TJUH) for further hematologic work-up.

In November of 2018, ~6 months prior to admission to TJUH, the patient received routine outpatient lab-work showing a platelet count of 547,000 cell/mcL. At time of formal hematological evaluation, her platelet count had decreased to 446,000 cell/mcL. She had no evidence of iron deficiency anemia or positive markers for inflammation (Table 1). PCR analysis for the JAK2 V617F point mutation was positive. She had a presumed diagnosis of ET, although she did not have a bone marrow biopsy at the time of diagnosis (10). Hydroxyurea was not started. Repeat lab work in April of 2019, ~1 month before admission to TJUH, revealed a platelet count of 262,000 cells/mcL and white cell count of 14,770 cells/mcL (48.1% lymphocytic). No plasma cells were noted on the differential.

**Table 1:** Temporal comparison of lab work from November 2018 (Nov-18), December 2018 (Dec-18), April 2019 (April-19), and May 2019 (May-19)—admission to Thomas Jefferson University Hospital.

**Variable**	**Ref range**	**Nov-18**	**Dec-18**	**April-19**	**May-19**
**Blood**					
White blood cell count (×10^3^/mcL)	4.0–11.0	5.8	6.89	14.77	60.9
Neutrophils (%)-Auto	49–80	50.6	48.3	21	
Lymphocytes (%)-Auto	13–43	32.9	35.7	48.1	
Monocytes (%)-Auto	0.0–10	12.9	12.2	27.6	
Basophils (%)-Auto	0.0–1.5	0.7	0.6	0.9	
Eosinophis (%)-Auto	0.0–0.6	2.9	2.8	2	
Plasma Cells (%)-Auto	0	0	0	0	
Immature (%)-Auto	0.0–0.5	0	0.4	0.4	
Neutrophils (%)-Manual	40–73				12
Lymphocytes (%)-Manual	20–44				22
Monocytes (%)-Manual	0–13				3
Basophils (%)-Manual	0–3				1
Eosinophils (%)-Manual	0–6				0
Plasma Cells (%)-Manual	0				60
Red-cell count (×10^6^/mcL)	3.7–5.3	4.19	4.06	3.89	3.02
Hemoglobin (g/dL)	11.5–16.1	13.4	13.1	12.5	9.6
Hematocrit (%)	34–47.5	39.5	39.6	36.8	28.8
Mean corpuscular hemoglobin (pg)	28–35	32.0	32.3	32.1	31.8
Mean corpuscular volume (fL)	81–99	94.3	97.5	94.6	95
Platelet count (×10^3^/mcL)	140–400	547	446	262	190
Iron (mcg/dL)	50–170		74		
Iron binding capacity total (mcg/dL)	250–450		298		
Ferritin (ng/mL)	8–252		65		
C reactive protein (mg/dL)	0.0–0.9		<0.2		
Sedimentation rate (mm/hr)	0.0–30		12		

Given her mild leukocytosis in April of 2019, her physician ordered a peripheral blood flow cytometry. The phenotypes shown were a mixed population of maturing myeloid cells, monocytes, eosinophils, B cells, and T cells. CD34 positive blasts comprised 0.1% of the total nucleated cells. Monocytes and eosinophils comprised 4.7 and 1.6%, respectively. There was no presence of a clonal plasma cell population (CD38 cell population).

One day prior to transfer to TJUH, in May 2019, the patient presented to an outside hospital after a mechanical fall involving her walker, resulting in a fracture of the left clavicle. A complete blood cell count upon admission to TJUH showed a white cell count of 60,900 cells/mcL with a manual differential indicating 60% plasma cells: ~36,540 plasma cells/mcL (36.5 × 10^9^/L), well above 2 × 10^9^/L plasma cells as per PCL diagnostic criteria (Table 1). Peripheral blood flow cytometry showed a monoclonal plasma cell population, comprising 70% of analyzed cells showing the following phenotype: CD5−, CD10−, CD19−, CD20−, CD22−, CD38+ (bright), CD138+, CD45−, CD56−, CD117−, cytoplasmic kappa light chain+ (dim), and cytoplasmic lambda light chain-. On bone marrow examination, the aspirate smear showed sheets of plasma cells comprising 95% of all nucleated cells with an overall hypercellular marrow of 90%. Of note, rare megakaryocytes were seen. Fluorescence *in-situ* hybridization (FISH) analysis revealed that the plasma cells were positive for loss of centromere 7 (7q), typical and atypical translocations of CCND1/IGH [(11;14)(q13.3;q32.3)], rearrangement of IGH gene involving 14q32.3, deletion of RB1 (13q), and deletion of TP53 (17p); the MPN FISH panel on non-plasma cells was positive for deletion 5q, loss of centromere 7 (7q), deletion of BCR (22q), and deletion of Rb1 (13q) (Table 2).

**Table 2:** Fluorescence *in-situ* Hybridization (FISH) result summary: including multiple myeloma (MM) FISH panel on C138+ enriched plasma cells and myeloproliferative neoplasm (MPN) panel on non-enriched unstimulated non-plasma cells.

**Positive findings for the CD138+ enriched MM fish panel**	**Positive findings for the MPN panel**
Positive for loss of centromere 7 (CEP 7)	Positive for deletion 5q−76%
Positive for typical and atypical translocations of CCND1/IGH	Positive for loss of centromere 7 (CEP7)−58%
Positive for rearrangement of IGH	Positive for deletion BCR (22q)−72.5%
Positive for deletion RB1 (13q)	Positive for deletion RB1 (13q)−71.5%
Positive for deletion TP53 (17p)	Negative for trisomy 8
Negative for deletion of 1p and gain of 1q	
Negative for trisomy 7, trisomy 9, and trisomy 11	
Negative for rearrangement of MYC	

*Percentages for MPN panel findings indicate percentage of nuclei positive for deletion*.

**Figure 1:**
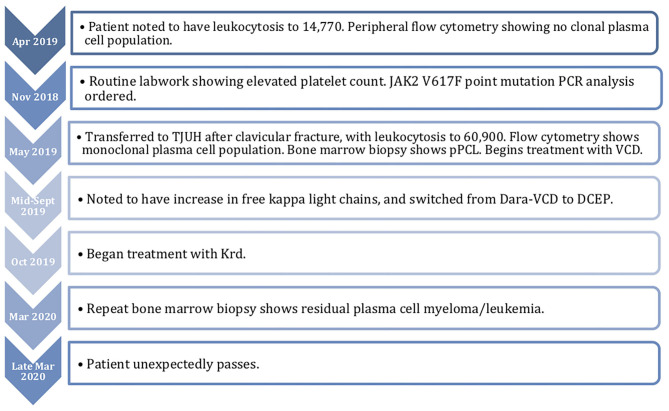
Timeline of patient's course.

In late May 2019, the patient underwent treatment with 1 cycle of bortezomib, cyclophosphamide, and dexamethasone (VCD). This was complicated by an admission for volume overload. Her white blood cell count fell from 80,000 to 50,000 during the first few days of treatment, but it was not completely controlled. She was then switched to Dara-VCD. During cycle 1, she was admitted to the hospital for renal failure, during which she became dialysis dependent. After 4 cycles of Dara-VCD, her free kappa light chains decreased from 18,014 mg/L to 8,394 mg/L by September 2019. She was ultimately taken off dialysis. In mid-September, her free kappa light chains increased to 9,396, requiring initiation of cycle 1 of dexamethasone, cyclophosphamide, etoposide, and cisplatin (DCEP). This was performed inpatient, and her free kappa light chains decreased to 4305.2 mg/L. She then began treatment with carfilzomib, lenalidomide, and dexamethasone (Krd) in October 2019. During cycle 2, carfilzomib was lowered to 56 mg/m^2^ weekly. This cycle was delayed due to admission for neutropenic fever. During initiation of cycle 3, her free kappa decreased to 639 mg/L. For cycle 4 in February 2020, her revlimid was held temporarily, but restarted on day 11. She also had an admission for pneumonia. Her free kappa light chains at this time were 774 mg/L. One year after the patient's initial presentation, the smear from a repeat bone marrow examination in March 2020 showed residual plasma cell myeloma/leukemia (~10% marrow involvement). JAK2 V617F mutation analysis was positive. She unfortunately unexpectedly passed in late March 2020 while at home. A timeline of her course is detailed in Figure 1.

## Diagnostic Assessment

### JAK2 Mutation Analysis

The laboratory test was performed at Integrated Oncology, a business unit of Esoterix Genetics Laboratory, LLC, a wholly owned subsidiary of Laboratory Corporation of America Holdings. Genomic DNA was extracted and amplified by real-time PCR using primers for the appropriate region of the JAK2 gene. The mutant and wild-type JAK2 sequences were detected using specific fluorescent probes. The analytical sensitivity of the assay is 1%: detection of 1 mutant copy of the JAK2 gene per 100 normal copies.

### Fluorescence *in-situ* Hybridization (FISH) Study: MM and MPN Panels

The MM FISH panel study was performed on CD138+ enriched plasma cells; the MPN FISH study was performed on non-enriched unstimulated cells, results of which likely represent abnormalities from non-plasma cells or a mixture of different cell populations. Probe sets from CytoTest, Inc. The cutoff value for the 5q, 7q, BCR/ABL1 translocation, RB1, 17p13.1, 20q12, IGH rearrangement, CCND1/IGH translocation, and MYC gene rearrangement were <5, <6, <2, <8, <8, <7, <7, <2, and <4%, respectively.

There were no overt diagnostic challenges in this case.

## Discussion

To our knowledge, this case is one of the few known reports of a patient with a concomitant MPN and pPCL. The literature of reported cases of patients with concomitant MPN and plasma cell dyscrasias raises much interest, namely because these malignancies originate from two separate cellular lineages in hematopoiesis.

Given the literature demonstrating this phenomenon, it has been proposed that in these instances of concomitant malignancies, a common cellular origin might exist. Wang et al. showed that both myeloid and lymphoid cells recovered from BM and spleens of mice with myelofibrosis both had positive JAK2 V617F mutations, suggesting the mutation occurred initially in a common hematopoietic stem cell (HSC) (11). Some evidence suggests that CML hematopoietic stem cell ancestry is shared with lymphocyte stem cell ancestry of the B-cell lineage (12). In addition, acute leukemia can be of mixed lineage origin or even undergo lineage switch suggesting a common hematopoietic stem cell (13). Hou et al. had found a novel human B cell/myeloid common progenitor cell based on absence of CXC chemokine receptor expression reinforcing the concept of shared ancestry (14). A case of a woman with chronic lymphocytic leukemia (CLL) and JAK-2 V617F mutation was reported showing that the JAK2 mutation existed in the B lymphocytes, but not in T lymphocytes. In this case, it was postulated that the JAK-2 mutation was a secondary event to which a primary gene mutation occurred at the common B lymphoid and myeloid stem cell—further suggesting the possibility of a common, pluripotent, myeloid, and B lymphoid progenitor cell (15). Another report assessing the presence of JAK2 V617F mutation in different cell lines in eight patients with PV found that six of these patients solely had the JAK2 mutation in the myeloid lineage of cells; however, one PV patient was found to have the mutation present in both B and T lymphocytes (as well as the myeloid cells), while another PV patient was found to have the mutation present in the B lymphocytes in addition to myeloid cells (16). This indicates that the JAK2 mutation can occur as early as the multipotent HSC level in some MPN cases.

On the FISH analysis utilizing a MM panel on the patient's plasma cells (Table 2), certain common chromosomal abnormalities were present that are common in pPCL. Common pPCL chromosomal abnormalities present included IgH translocations, loss of 13q, and loss of 17p (TP53). For the MPN FISH panel conducted, the deletion of 5q, 7q, and 13q are chromosomal abnormalities notable in MPNs; 13q deletion was also seen in the plasma cells, which is a deletion also found to be prevalent in CLL, MM, and pPCL, as previously mentioned (17). The positive deletion of 13q in the MPN panel is less common for typical MPN chromosomal abnormalities (17). The shared abnormalities on the FISH panel for the plasma cells and non-plasma cells included the 13q deletion and 7q deletion. Interestingly, the 7q deletion is not a typical finding in pPCL FISH analysis, since it is more associated with myeloid disorders (18, 19). The shared 7q deletion suggests that the patient's MPN and plasma cell leukemia may have arisen from a common cellular origin. The 7q deletion, despite being a predominantly myeloid chromosomal abnormality, is also a prognosticator of poor outcome, especially for leukemic transformation in MPN (20, 21).

There have, however, been studies published that argue against the theory of a common cellular origin for these two malignancies. Kuroda et al. showed independent genetic mutations in a patient with the coexistence of multiple myeloma and ET; the JAK2 V617F was identified in peripheral white blood cells and bone marrow-derived non-myeloma cells, but not in CD138+ myeloma cells from the bone marrow (22). Additionally, despite previously stated, there have been reports of patients with concurrent CLL and JAK2 V617F positive MPN that did not show the JAK2 mutation present in the lymphoid cells (4). Overall, it appears there is mixed evidence in support of a common cellular lineage for when these two separate malignancies appear in the same patient; however, it remains possible that in some instances of concomitant MPNs and plasma cell dyscrasias, there might be a common cellular origin.

It is also possible that the bone marrow microenvironment (BMM) could influence concomitant disease. Schofield first demonstrated that bone marrow cells transplanted from wild-type mice into W/Wv mice (mutation in kit) could continue hematopoiesis indefinitely. In comparison, cells that formed colonies in the spleen (colony-forming units-spleen cells) and were subsequently transplanted lost that ability (23, 24). While early studies focused on the role of the microenvironment in promoting normal hematopoiesis, more recent data has shown that alterations in the BMM may support malignant conditions including MPNs and myeloid leukemia (25–27). Lundberg et al. found that bone marrow vascularity was increased and disorganized in patients with MPN and correlated with JAK-2 burden (28). The endothelial cells of the BMM can also be stimulated by proangiogenic and pro-inflammatory factors to release VEGF, which causes increased blast survival and proliferation (29, 30). Plasma cell interactions with the BMM are not as well-studied, but there is evidence that various factors secreted by endothelial cells and stromal cells affect myeloma cell migration (26). While there are no specific studies in relation to the BMM and concomitant disease from a myeloid progenitor and lymphoid progenitor, it appears not unlikely that these individually studied phenomena could coexist in the described patient.

Speculation for the incidence of coexistence for these two malignancies has also been focused on other shared precipitating factors. Interleukin-6 (IL-6) has been postulated as a possible culprit in certain instances linking the association of these two malignancies. IL-6 is known as a promoter of platelet production as well as being a player in the pathogenesis of MM, given the fact that it is a potent human myeloma cell growth factor (4). Radiation or drug exposure, namely hydroxyurea, has been postulated as another shared precipitating factor for both malignancies, although not relevant in this particular case.

Strengths of this case report include its novelty, the availability of patient data and ability to assess this patient's outcome, and its educational value. While there is a similar case detailed from 2004 of a patient from 1997, there is still value in reporting this case, which is more than 15 years later. The patient detailed by Candoni et al. had previous exposure to cytoreductive therapy (hydroxyurea and busulphan), which may have contributed to the development of PCL. Our patient was not started on any similar treatment prior to her diagnosis of PCL. Further research is needed in the association of these diseases to understand whether these two cases reflect similar or divergent mechanisms of pathogenesis. Limitations included the lack of literature of this exact case given its novelty, which leads to an inability to generalize. While previous associations between MPNs and plasma cell dyscrasias have been noted in the literature, this is one of the few published reports of a case of a concomitant JAK-2 positive MPN and primary plasma cell leukemia. The exact mechanism by which this phenomenon occurs remains under debate due to their distinct lineages. As awareness of these cases grows, our hope is that the understanding of these disease states will be further elucidated.

## Data Availability Statement

All datasets generated for this study are included in the article/Supplementary Material.

## Ethics Statement

At time of writing of the manuscript the individual in the case described had passed away. Written informed consent was obtained from the individual's healthcare proxy and next of kin for the publication of any potentially identifiable images or data included in this article.

## Author Contributions

CK organized and wrote manuscript. CT and SH helped with writing of manuscript. TK and AB reviewed and edited manuscript. All authors contributed to the article and approved the submitted version.

## Conflict of Interest

The authors declare that the research was conducted in the absence of any commercial or financial relationships that could be construed as a potential conflict of interest.
