# Supplementary material for: Case Report: Concomitant Diagnosis of Plasma Cell Leukemia in Patient With JAK2 Positive Myeloproliferative Neoplasm
Source: Front Oncol. 2020 Aug 27;10:1497. doi: 10.3389/fonc.2020.01497 (PMC7482648; doi:10.3389/fonc.2020.01497)
Supplement: Supplementary file 1 [file Data_Sheet_1.PDF]

CARE case report guidelines

CARE Checklist of information to include when writing a case report

(cc) BY-NC-ND

Topic	Item	Checklist item description	Reported on Line
Title	1	The diagnosis or intervention of primary focus followed by the words "case report"	1
Key Words	2	2 to 5 key words that identify diagnoses or interventions in this case report, including "case report"	10
Abstract (no references)	3a	Introduction: What is unique about this case and what does it add to the scientific literature?	17-20
	3b	Main symptoms and/or important clinical findings	20-22
	3c	The main diagnoses, therapeutic interventions, and outcomes	23-24
	3d	Conclusion—What is the main "take-away" lesson(s) from this case?	23-24
Introduction	4	One or two paragraphs summarizing why this case is unique (**may include references**)	27-46
Patient Information	5a	De-identified patient specific information.	49-54
	5b	Primary concerns and symptoms of the patient.	49-54
	5c	Medical, family, and psycho-social history including relevant genetic information	49-54
	5d	Relevant past interventions with outcomes	Not applicable
Clinical Findings	6	Describe significant physical examination (PE) and important clinical findings.	55-71
Timeline	7	Historical and current information from this episode of care organized as a timeline	276
Diagnostic Assessment	8a	Diagnostic testing (such as PE, laboratory testing, imaging, surveys).	101-114
	8b	Diagnostic challenges (such as access to testing, financial, or cultural)	116
	8c	Diagnosis (including other diagnoses considered)	119
	8d	Prognosis (such as staging in oncology) where applicable	Not applicable
Therapeutic Intervention	9a	Types of therapeutic intervention (such as pharmacologic, surgical, preventive, self-care)	83-100
	9b	Administration of therapeutic intervention (such as dosage, strength, duration)	83-100
	9c	Changes in therapeutic intervention (with rationale)	83-100
Follow-up and Outcomes	10a	Clinician and patient-assessed outcomes (if available)	83-100
	10b	Important follow-up diagnostic and other test results	83-100
	10c	Intervention adherence and tolerability (How was this assessed?)	83-100
	10d	Adverse and unanticipated events	83-100
Discussion	11a	A scientific discussion of the strengths AND limitations associated with this case report	184-186
	11b	Discussion of the relevant medical literature **with references**.	119-191
	11c	The scientific rationale for any conclusions (including assessment of possible causes)	119-191
	11d	The primary "take-away" lessons of this case report (without references) in a one paragraph conclusion	184-191
Patient Perspective	12	The patient should share their perspective in one to two paragraphs on the treatment(s) they received	Not applicable
Informed Consent	13	Did the patient give informed consent? Please provide if requested	Yes ☑ No ☐
